# Supplementary material for: Association between Cerebrospinal Fluid and Serum Biomarker Levels and Diagnosis, Injury Severity, and Short-Term Outcomes in Patients with Acute Traumatic Spinal Cord Injury
Source: Diagnostics (Basel). 2023 May 22;13(10):1814. doi: 10.3390/diagnostics13101814 (PMC10217493; doi:10.3390/diagnostics13101814)
Supplement: Supplementary file 1 [file diagnostics-13-01814-s001.zip › diagnostics-2289198-supplementary.pdf]

Table S1. Correlation between CSF biomarker (SBDP150, UCH-L, S100B, Tau, GFAP, NF-L, IL-6) day one concentration patient with SCI and healthy control. *p values < 0.05 were considered statistically significant. AUC – area under the curve. CI – confidence intervals.

ROC of CSF biomarkers control vs SCI			
**Biomarkers**	**AUC**	**95% CI**	***P**
**SBDP150**	0.9036	0.7766 – 1.031	0.0009453
**S100b**	0.9357	0.8129 – 1.059	0.0003579
**IL-6**	0.9286	0.8120 – 1.045	0.0004466
**GFAP**	0.9231	0.7782 – 1.068	0.0006525
**NFL**	0.9769	0.9239 – 1.030	0.0001220
**UCHL-1**	0.7846	0.5823 – 0.9869	0.02179
**Tau**	0.8077	0.6230 – 0.9924	0.01315

Table S2. Correlation between serum biomarker (SBDP150, UCH-L, S100B, Tau, GFAP, NF-L, IL-6) concentration and diagnosis correlations in patients with SCI. *p values < 0.05 were considered statistically significant. ROC= receiver operating characteristic AUC= area under the curve. CI = confidence intervals.

**ROC of serum biomarkers control vs SCI**			
**Biomarkers**	**AUC**	**95% CI**	***P**
**SBDP150**	1.000	1.000 – 1.000	< 0.0001
**S100b**	0.8100	0.6293 – 0.9907	0.009927
**IL-6**	1.000	1.000 – 1.000	< 0.0001
**GFAP**	0.9643	0.8901 – 1.038	0.0006525
**NFL**	1.000	1.000 – 1.000	< 0.0001
**UCHL-1**	0.9400	0.8537 – 1.026	0.0002538
**Tau**	0.9400	0.8520 – 1.028	0.0002538

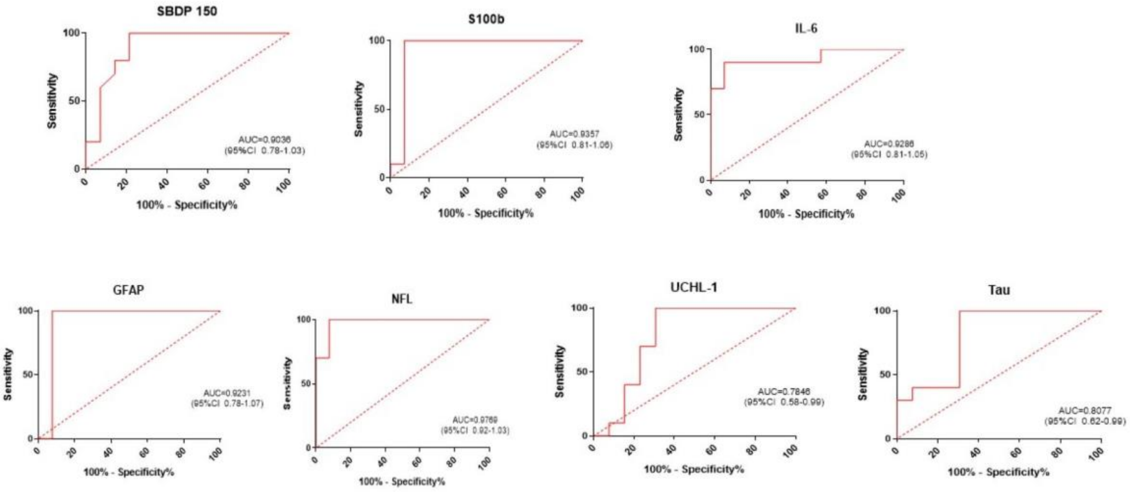

Figure S1. ROC curves for Day 1. CSF biomarkers for SCI patients versus controls for predicting the diagnosis of SCI.

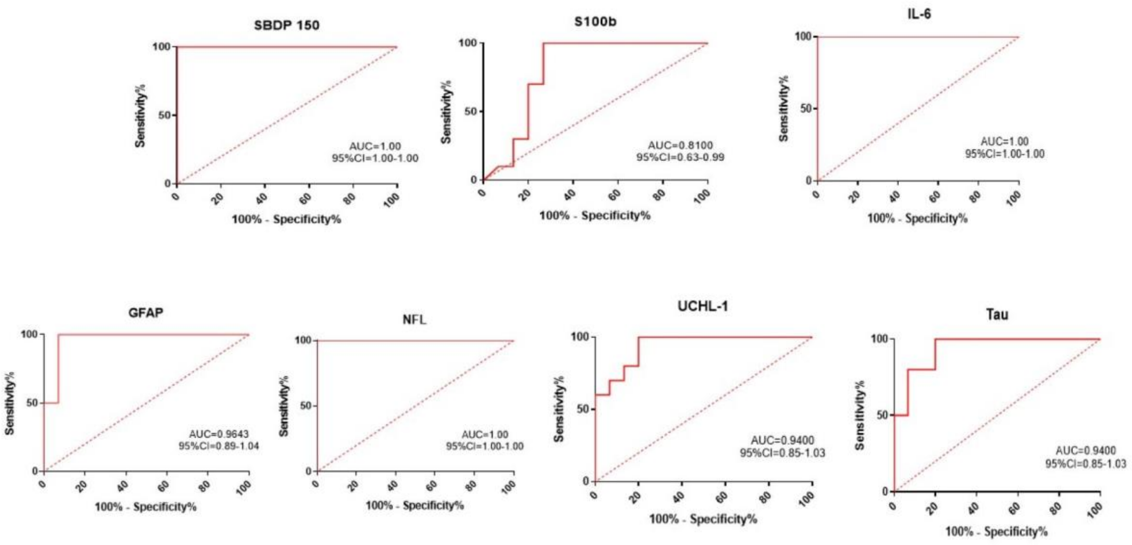

Figure S2. ROC curves for Day 1. Serum biomarkers for SCI patients versus controls for predicting the diagnosis of SCI.
